# Working Memory Workload When Making Complex Decisions: A Behavioral and EEG Study

**DOI:** 10.3390/s24175754

**Published:** 2024-09-04

**Authors:** Michela Balconi, Katia Rovelli, Laura Angioletti, Roberta A. Allegretta

**Affiliations:** 1 International research center for Cognitive Applied Neuroscience (IrcCAN), Università Cattolica del Sacro Cuore, 20123 Milan, Italy; michela.balconi@unicatt.it (M.B.); katia.rovelli@unicatt.it (K.R.);; 2 Research Unit in Affective and Social Neuroscience, Department of Psychology, Università Cattolica del Sacro Cuore, 20123 Milan, Italy

**Keywords:** decision-making, working memory, job assessment, EEG, metacognition

## Abstract

Working memory (WM) is crucial for adequate performance execution in effective decision-making, enabling individuals to identify patterns and link information by focusing on current and past situations. This work explored behavioral and electrophysiological (EEG) WM correlates through a novel decision-making task, based on real-life situations, assessing WM workload related to contextual variables. A total of 24 participants performed three task phases (encoding, retrieval, and metacognition) while their EEG activity (delta, theta, alpha, and beta frequency bands) was continuously recorded. From the three phases, three main behavioral indices were computed: Efficiency in complex Decision-making, Tolerance of Decisional Complexity, and Metacognition of Difficulties. Results showed the central role of alpha and beta bands during encoding and retrieval: decreased alpha/beta activity in temporoparietal areas during encoding might indicate activation of regions related to verbal WM performance and a load-related effect, while decreased alpha activity in the same areas and increased beta activity over posterior areas during retrieval might indicate, respectively, active information processing and focused attention. Evidence from correlational analysis between the three indices and EEG bands are also discussed. Integration of behavioral and metacognitive data gathered through this novel task and their interrelation with EEG correlates during task performance proves useful to assess WM workload during complex managerial decision-making.

## 1. Introduction

### 1.1. Background

Organizations often rely on the decisions of their front-lines managers, and this underlines how decision-making ability is a crucial skill to evaluate and train professionals, in order to help them choose the best options for the company as well as enhance the motivation and performance of the people they supervise [1]. Indeed, according to Wood and Bailey [2], managerial decision-making takes place in a continuous stream of activity in which choices made at one moment in time affect the possibilities and outcomes of decisions made at a later time. Additionally, Samson and Bhanugopan [3] stated that the effectiveness of managerial decision-making directly influences how much strategic human capital analytics enhance organization and market performance.

Accordingly, in such a complex and dynamic environment, effective decision-making requires individuals’ cognitive functions to be continuously engaged to gather information, evaluate feedback, test, revise knowledge, and execute selection possibilities [1]. Moreover, it has been recently argued that for an effective decision-making process and to respond appropriately to workplace demands good executive functioning is necessary [4]. Executive functions (EFs) are a group of top-down mental operations that include inhibition (self-control and interference control), working memory (WM), and mental flexibility, promoting goal-directed behavior [5,6]. These functions are also considered essential for sustained attention, control of automatic responses, as well as quick and flexible responses to the environment’s changing demands [7,8].

Among the other EFs, scholars have suggested that WM is one of the most crucial functions for an adequate performance execution [9]. In fact, good WM functioning can help a person to focus on the components of a current situation and be better able to remember previous experiences to recognize patterns and connect disparate information [9]. WM is essential because it organizes information that is no longer perceptually present [10] and enables decision-makers to access both repertoires of potential solutions to classes of issues that have previously been encountered as well as repertoires of problem–solution components [11].

Classically, WM has been defined by Baddeley [12,13] as a system that serves as a bridge between attention, perception, and action, momentarily storing information and facilitating thinking. More recently, Diamond [6] stated that WM serves as an “attention buffer” and depends on two main processes: actively processing concepts while holding them in mind. Thanks to these two mechanisms, WM enables people to pay attention to various elements and recognize patterns even in the face of uncertainty. On the adaptive level, it allows for the creation and retention of an internal representation of relevant information that can be applied later to guide behavior [14].

Usually, in cognitive psychology and neuropsychology, WM is tested and evaluated with classical laboratory-based tasks, such as the Listening Span Test [15], word or number span, or N-back task [16], which can be useful as a pure performance measure, but are less ecological for applied contexts, such as organizations. To fill this gap and evaluate the WM contribution to managerial decision-making, it proves necessary to design novel ecological tasks that allow gathering behavioral data during the whole managerial decision-making process (including the encoding and retrieval phases, as well as the participant’s awareness of the decision).

Additionally, useful tools, which can allow the recording and monitoring of the neural activity supporting the cognitive processes adopted in the decision-making process, can be provided by neuroscience and especially by the neuromanagement discipline, which strives to investigate and assist changes within organizations by applying neuroscience methodologies and approaches to management [17,18] and focuses on the differentiation between managers’ explicit and implicit attitude by operationalizing and tracing implicit attitude with specific neuroscientific techniques [19].

In particular, between the other neuroscientific tools, electroencephalography (EEG) allows the recording of brain electrophysiological activity in real-time and with a high temporal resolution. Specifically, EEG is a non-invasive technique that, through electrodes placed on the scalp, can record slight fluctuations in electrical potential generated by the activity of cortical neurons, by a temporal resolution ranging from milliseconds; in addition, this instrument can be used with great advantages also as a wearable device—thus, with its “self-fitting characteristic”, it can be easily applied by the subjects themself [20]—in a non-laboratory setting such as organizations, as shown by several recent studies [21,22,23].

Also, through the analysis of frequency bands (delta, theta, alpha, and beta), as well as through the analysis of their localization on the scalp and their functional meaning, it is possible to study and investigate brain activity linked to specific perceptual, cognitive, and emotional processes. Getting down to the specifics, the alpha band is mostly associated with the inhibition of processes that are considered conflicting or useless for the task proposed to the individual [24]. Regarding its localization, alpha rhythm is mostly produced over posterior brain regions [25,26]. Also, the increase in alpha activity in specific brain locations may also suggest that a targeted area is not crucial for information processing and its activity is, instead, suppressed while other brain regions are more involved in the process [27,28]. On the other hand, beta waves increase with attention and vigilance, and this may reflect an increase in excitatory activity, particularly in states of arousal or focused attention and cognitive processing [29,30,31]. In several visual WM studies, beta has been observed in posterior areas [32,33,34]. Instead, theta oscillations are characterized by a median frontal distribution and are involved in attentional and memory processes [35,36] as well as in the processing of emotional responses [4], also in social decision-making contexts. The anterior cingulate cortex has been repeatedly indicated as one of the generators of this activity [29]. Finally, delta waves have been interpreted as a correlate of cortical inhibition, but it has also been shown that the event-related increase in delta power can be considered a correlate of active cognitive processes [37], explicit and declarative memory formation, and emotion states [38,39,40].

Moreover, it is worth noticing that several studies combined an EEG and behavioral approach to monitoring WM load under stress [41,42,43], showing an interrelation between behavioral task performance and the EEG pattern of brain activity. Indeed, former studies showed how optimal behavioral performance may be predicted based on neural dynamics [27,44]. For instance, alpha band desynchronization was demonstrated to be an oscillatory indicator of WM load, showing a plateau at difficulty levels surpassing WM capacity [45]. Also, some evidence points to a positive correlation between beta band activity and WM load [46], while other scholars demonstrated a decreased beta power associated with high cognitive WM load [47,48,49].

### 1.2. Related Studies

Therefore, taking advantage of the combination of behavioral and EEG data, the present work investigated the behavioral and EEG correlates of healthy individuals performing a novel WM task that strives to measure WM workload in relation to contextual variables, using real-life ecological situations related to managerial decisions. Also, the individuals’ ability to consciously self-represent the challenges encountered throughout the task, as well as their ability to tolerate and act in situations of growing decisional complexity was assessed.

The ecological features of the task were of particular importance, since, in the organizational context, a classic WM task that uses numbers, letters, or words may not be appropriate to reliably measure the WM workload conditions to which managers and professionals are subjected. Indeed, they must face daily complex situations that require the integration of contextual variables and knowledge of the skills of their employees, to assign them a specific work assignment.

Thus, the task proposed in this study was composed of three phases: (i) an encoding phase, in which participants were presented with ten profiles of professional employees that they needed to memorize in 5 min; (ii) a retrieval phase, where six scenarios—representing relevant work assignments—were showed on the screen and participants were asked to select the employee they believed would be most capable of carrying out the work outlined with the greatest efficiency (this phase presented an incremental difficulty due to the increasing number of profiles to assign within each scenario); and (iii) a metacognitive phase, participants were asked to complete a metacognitive scale reflecting the perceived grade of difficulty encountered during each step of the decisional process.

During the encoding and retrieval phase, an EEG was recorded continuously to collect the EEG correlates (in terms of frequency band variations) associated with the WM load. Also, from these three phases, different behavioral indices, based on participants’ responses and response times (RTs), were computed to measure participants’ WM load under stress.

Firstly, the index of Efficiency in complex Decision-making is determined from the number of correct responses to the employee’s assignment task and the relative RTs—which can provide a measure of how efficiently a person can correctly respond during the retrieval phase in the shortest time. This index also reflects the encoding phase; indeed, when facing an increasingly complex task, the best way to successfully complete it is to use an appropriate strategy starting from the elaboration and memorization of available information (i.e., from the encoding phase). In fact, with strategic thinking managers foresee the outcomes of their activities while deciding which actions to take and analyze the impacts of their decisions to assess the effectiveness of their efforts [1].

Secondly, the index of Tolerance of Decisional Complexity was obtained from the number of completed scenarios during the retrieval phase weighted over the efficiency in the complex decision-making index. This index can provide a measure of how the person was able to make a correct decision while tolerating increased complexity and ambiguity; ambiguity tolerance has been considered by scholars an essential ability for making high-quality complex decisions and correlates with self-efficacy.

Thirdly, the index of Metacognition of Difficulties provides the individual’s perceived level of difficulty while accomplishing the task. In fact, it is also essential to have a proper representation of the challenges encountered during the decision and of one’s own competencies that enable the evaluation and prediction of the action’s outcome (self-monitoring functions) [50,51].

Based on these theoretical and methodological premises, we formulated the following hypothesis.

First, we hypothesize that alpha and beta bands might have a central role both during the encoding and retrieval phase. Since an increased alpha band has been widely associated with inhibition of task interfering processes [24], we expected to find a decreased alpha activity indicating high cognitive engagement [52] during both the encoding and retrieval phases. In particular, during the encoding phase this activation was expected over temporoparietal regions that have been associated with verbal WM (VWM) and fundamental language functions [53,54] during VWM performance; meanwhile, during the retrieval phase the decrease over the same areas might indicate retrieval of semantic integrated information [27,55] and active information processing [28,56].

Concerning the beta band, in line with recent findings [57], we expected to observe a decrease in the beta band over temporoparietal areas (e.g., right temporal gyrus) [32] during the encoding phase, indicating a load-related effect, sustained attention, and a maintenance process. On the contrary, during the retrieval phase, we expect to find an increase in beta oscillation over posterior areas, related to increasing WM task complexity [46,58] and filtration of irrelevant information for task resolution.

Thirdly, with reference to the link between behavioral indices and EEG data, it was expected that we would observe the relation between the index of Efficiency in complex Decision-making and EEG, with specific reference to theta and alpha bands. Indeed, theta activity might increase in frontal areas as a response to a socio-emotional situation [39,59]—which might be elicited by the real-life decision-making situation described by the task—as well as WM processing [60] and attention, while decreased alpha activity over the same areas might indicate high cognitive activity [52].

Also, a correlation between the index of Tolerance of Decisional Complexity and the beta band was also expected during the encoding phase; that is, task complexity might elicit a state of arousal as well as focused attention and cognitive processing [29,30,31].

Finally, we expected to observe a relation between the index of Metacognition of Difficulties and the delta band, indicating an emotional state linked to and increased by the evaluation of the person’s perceived level of difficulty during the performance, as well as an effort to concentrate and recall the performance in order to evaluate it.

## 2. Method

### 2.1. Sample

This study recruited a sample of 24 Italian professional participants (Mage = 35.33, SDage = 11.94, age range: 23–60, Nmale = 13, Nfemale = 11). The inclusion criteria encompassed the following aspects: individuals with either normal vision or vision corrected to normal and individuals with either normal hearing or hearing corrected to normal. The following conditions were taken into consideration as exclusion criteria: clinically significant distress; history of neurologic or psychiatric illnesses; serious head injury and/or ictus; and current therapy based on psychoactive substances that could affect decision-making or cognition. No remuneration was provided to the participants in this study and each participant provided written informed consent.

The assessment process and the study were developed in accordance with the 2013 revision of the Declaration of Helsinki and according to the GDPR–Reg. UE 2016/679 and its ethical guidelines. The Ethics Committee of the Psychology Department of the Catholic University of The Sacred Heart, Milan, Italy, approved the research protocol.

### 2.2. Procedure

The participants were briefed on the experimental setup and procedures in a quiet room. Participants were provided with an explanation of the experiment and were asked to provide their informed consent through a signed document. Subsequently, they were instructed to take a stationary position, sitting approximately 80 cm away from a high-resolution computer monitor placed on a desk. Before the experimental task, wearable dry EEG systems were applied to participants and a 120 s of resting state baseline was collected.

**Figure 1:**
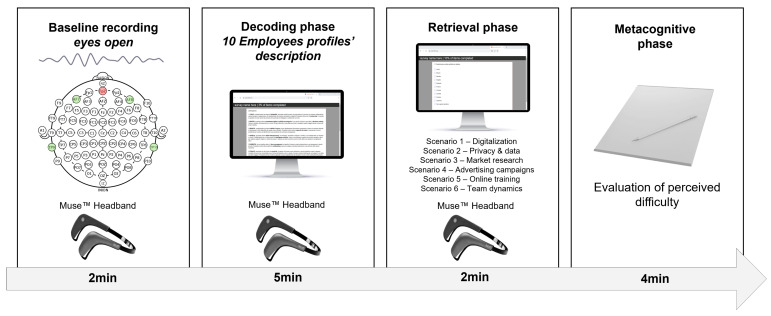
Experimental procedure. The figure shows the procedural flow illustrating the three phases of the task: encoding, retrieval, and metacognition phase.

Upon the completion of the preparatory phase, participants were then informed with task instructions. Subsequently, they performed the task while EEG data were continuously recorded for the encoding and retrieval phase of the task. The comprehensive process encompassed approximately 30 min (Figure 1).

### 2.3. Experimental Task

In this study, participants were asked to perform a novel managerial task developed to investigate the WM workload and subjects’ awareness of their choices, in relation to the degree of decisional complexity, through a web-based survey and experiment management platform (PsyToolkit) [61,62]. The task was divided into three main phases: (i) the encoding, (ii) retrieval, and (iii) metacognitive phase.

#### 2.3.1. Encoding Phase

The encoding phase required participants to memorize ten different professional profiles of employees within a company within a limited time frame (five minutes). During this initial phase, participants were informed of the task’s instructions and were made aware that, in the subsequent phase, they would be presented with scenarios requiring the involvement of some of these employees. Their task would be to select, from the presented profiles, the employee that is best suited to efficiently perform each of the presented job assignments. See Table 1.

#### 2.3.2. Retrieval Phase

Subsequently, in the second phase of the task—the retrieval phase—participants were presented with six different scenarios (see Table 2).

The scenarios were selected based on ecological demands within the organizational management context to ensure a close approximation to the everyday real working environment, namely Digitalization (Scenario 1), Privacy and data (Scenario 2), Market research (Scenario 3), Advertising campaigns (Scenario 4), Online training (Scenario 5), and Team dynamics (Scenario 6).

Following the description of each scenario, there was a list of two or more work assignments consistent with the scenario, and the participant was required to choose, through multiple-choice and within a maximum time limit of 20 s, the most suitable employee from the list of ten profiles to fulfil the given work assignment. An additional option of “*I don’t know*” was also provided.

In total, 22 allocations were requested, specifically starting with two allocations for Scenario 1 (Digitalization) and Scenario 2 (Privacy and data), three allocations for Scenario 3 (Market research), four allocations for Scenario 4 (Advertising campaigns), five allocations for Scenario 5 (Online training), and escalating to six allocations for Scenario 6 (Team dynamics). For example, in Scenario 1 (Digitalization) two employee allocations were required: given the skills described in their profiles, Mauro was the correct answer for the first work assignment focusing on the design of the digital platform, and Caterina was the correct answer for the second work assignment focusing on the design of creative content.

Furthermore, along with the increasing challenge posed by the number of assignments required for each scenario, there is also a growing level of difficulty associated with the time pressure required to complete the task. In fact, the time for these allocations remained consistent, with a fixed duration of 20 s in total.

#### 2.3.3. Metacognitive Phase

Finally, the third phase of the experiment, the metacognitive phase, was designed to examine participants’ abilities to evaluate the entire cognitive processes during the managerial decision-making tasks. The aim of this phase was to delve into the cognitive processes, self-monitoring mechanisms, and self-regulatory strategies employed by individuals, thus furnishing a comprehensive perspective that not only encompasses the quality of decisions, but also metacognition as a requisite for adaptive and effective decision-making.

In this phase, they were asked to fill in a metacognitive scale that assesses the perceived level of difficulty experienced during each scenario of the task. A total of six items, corresponding to the six scenarios of the task, were presented one at a time to the participant. For each item, a five-point Likert scale, ranging from a minimal to a maximal level of difficulty, to comprehensively assess the completion of each of the scenarios (“For each step, indicate the degree of difficulty you encountered with a number from 1 to 5, where 1 indicates the least degree of difficulty and 5 the maximum degree of difficulty”). Participants had a maximum allowed time of 10 s to provide an answer to each single item.

### 2.4. Behavioural Data Acquisition and Behavioural Indices

Concerning behavioral data, participants’ responses and reaction times (RTs) for each scenario of the task were collected.

Specifically, RTs were collected as an indirect measure of cognitive load and the underlying processes of identification and decision-making. This methodological approach not only enabled us to gauge participants’ cognitive effort and workload under progressively demanding conditions but also aligned with existing research paradigms for the assessment of cognitive workload. It permitted an examination of how effectively participants could match available personnel resources to task requirements, shedding light on their capacity to manage complex decision-making scenarios within defined time constraints.

In the next sections, the behavioral indices computed from the combination of participants’ responses and RTs will be described. All behavioral indices were converted into deciles to compare the results derived from the different phases of the task.

#### 2.4.1. Index of Efficiency in Complex Decision-Making (EffDec_dec_–i)

To compute the index of *Efficiency in complex Decision-making* (EffDec_dec_–i*)* the following steps were followed.

First, a score of 1 was assigned for each correct allocation of employees in response to various allocation requests, while a score of 0 was assigned for each incorrect allocation of employees to a work assignment. In fact, for Scenario 1 (Digitalization) and Scenario 2 (Privacy and data) a maximum score of two (for each scenario) could be obtained. For Scenario 3 (Market research) a maximum score of three could be obtained. For Scenario 4 (Advertising campaigns) a maximum score of four could be obtained, as well as a maximum score of five for Scenario 5 (Online training) and of six for Scenario 6 (Team dynamics). The total score consisted of the sum of the correct allocations for all scenarios and could range from 0 to 22. It was likewise converted into a decile-based scale from 1 to 10, defining the index of *Performance in the human resource Allocation task* (PerfAll_dec_–i), for which higher scores corresponded to a better decision-making performance in terms of work assignment matching.

Secondly, for each scenario RTs were collected, averaged between all scenarios and converted to a decile-based scale from 1 to 10, defining the index of *Performance in the human resource Allocation task* RT (PerfAllRT_dec_–i).

Finally, the ratio between the two latter indices (PerfAll_dec_–i; PerfAllRT_dec_–i) composed the index of *Efficiency in complex Decision-making* (EffDec_dec_–i). Higher scores corresponded to a higher efficiency in complex decision-making. The index was computed as follows:

$${\mathrm{EffDec}}_{\mathrm{dec}}–i=\frac{{\mathrm{PerfAll}}_{\mathrm{dec}}–i}{{\mathrm{PerfAllRT}}_{\mathrm{dec}}–i}$$

#### 2.4.2. Index of Tolerance of Decisional Complexity (TolDecCom_dec_–i)

To compute the index of *Tolerance of Decisional Complexity* (TolDecCom_dec_–i) the following steps were followed.

First, a score of 1 was assigned to each successfully completed scenario, while a score of 0 was assigned if the participant did not complete the scenario or selected the option “I don’t know”. To consider a scenario completed, it was sufficient that at least one employee was correctly allocated to the specific work assignment proposed in the scenario. For example, in Scenario 1 (Digitalization) two employee allocations were required (Mauro was the correct answer for work assignment 1 and Caterina was the correct answer for job assignment 2), but it was sufficient that at least one employee (Mauro or Caterina) was correctly allocated to one of the work assignments to consider the scenario successfully completed. A total score, representing the number of completed scenarios for each participant and ranging from 0 to 6, was subsequently converted offline into a common scale expressed in deciles (from 1 to 10) in which comprised the index of *Tolerance of complex Decisions* (TolDec_dec_–i), for which higher scores corresponded to a higher ability to tolerate the complexity of the decision.

Subsequently, the index of *Tolerance of Decisional Complexity* (TolDecCom_dec_–i), expressing the subject’s ability to tolerate and make decisions in a situation of increasing decisional complexity, was computed as the mean of TolDec_dec_–i and the EffDec_dec_–i, as follows:

$${\mathrm{TolDecCom}}_{\mathrm{dec}}–i=\frac{{\mathrm{TolDec}}_{\mathrm{dec}}{–i+\mathrm{EffDec}}_{\mathrm{dec}}–i}{2}$$

#### 2.4.3. Index of Metacognition of Difficulties (MetaDiff_dec_–i)

Finally, for computing the index of *Metacognition of Difficulties* (MetaDiff_dec_–i) the following steps were followed.

As a first step, a Performance score based on correct responses and RTs was computed. For each scenario, the number of correct responses (Nhits_scenN_) over the maximum number of correct answers (Maxhits_scenN_) for that scenario was calculated. Then, the RT for responding to the scenario was divided by the maximum allowed time for the response (20 s). The formula for the Performance score is reported below:

$${\mathrm{Performance}}_{\mathrm{scenN}}=\frac{\frac{{\mathrm{Nhits}}_{\mathrm{scenN}}}{{\mathrm{Maxhits}}_{\mathrm{scenN}}}}{\frac{{\mathrm{RT}}_{\mathrm{scenN}}}{20}}$$

As a second step, a Metacognition score based on item responses and RTs was computed. In this case, the score obtained for the item on the metacognitive Likert scale, ranging from 1 to 5 points, (Metascore_item_) was divided by the maximum score allowed (5 points). The RT score to provide an answer to each item RT_item_ was divided by the maximum allowed time for the response (10 s). The formula for the Metacognition score is reported below:

$${\mathrm{Metacognition}}_{\mathrm{scenN}}=\frac{\frac{{\mathrm{Metascore}}_{\mathrm{itemN}}}{5}}{\frac{{\mathrm{RT}}_{\mathrm{itemN}}}{10}}$$

Subsequently, the Performance score was weighted over the Metacognition score to obtain the score of *Representation of Difficulties* (RepDiff_scenN_*)* for each scenario.

Finally, the index of *Metacognition of Difficulties* (MetaDiff_dec_–i), expressing the individual’s ability to consciously self-represent the difficulties in the decision-making process, was obtained as the average of the Representation of Difficulties scores for the six scenarios, as reported in the formula below:

$$\mathrm{MetaDiffdec}–i=\frac{{\mathrm{RepDiff}}_{\mathrm{scen}1}{+\mathrm{RepDiff}}_{\mathrm{scen}2}{+\ldots +\mathrm{RepDiff}}_{\mathrm{scen}6}}{N}$$

### 2.5. EEG Data Acquisition and Biosignal Analysis

A wearable dry-EEG device (Muse^TM^ headband, version 2; InteraXon Inc, Toronto, ON, Canada) was used to record EEG data. This system uses three medial frontopolar electrodes as a reference, and four electrodes for collecting the EEG signal from the prefrontal (AF7 and AF8) and temporoparietal (TP9 and TP10) regions. The frontal and temporoparietal electrodes on the Muse^TM^ headband are made of silicon rubber and are positioned in accordance with the 10/20 International System for electrode placement.

All data were recorded and collected through the mobile app “Mind Monitor” via Bluetooth transmission to a connected smartphone. Data were gathered at a fixed sampling rate of 256 Hz while being subjected to a 50 Hz notch frequency filter. Participants were instructed to limit the number of eye blinks and movements to reduce the presence of artifacts. For the purpose of calculating power density values for the standard EEG bands (delta, theta, alpha, beta, and gamma), frequency components of the EEG signals were extracted using an automated Fast Fourier Transformation approach. The recording of a 120 s baseline took place at the beginning of the experimental phase and, for each participant, EEG activity during the experimental conditions was weighted over baseline values.

Since the quality of data collected with a wearable dry-EEG device may be compromised by artifacts caused by the dry electrodes, which are prone to interference from eye blinks and muscle movements (such as jaw clench) [63], artifact rejection was initially performed using Principal Component Analysis (PCA). Subsequently, all data were visually inspected, and any remaining artifacts were manually removed.

### 2.6. Data Analysis

Data analysis was divided into two steps.

Firstly, four repeated-measure ANOVA with *Electrode* (4: AF7, AF8, TP9, and TP10) and the *Task Phase* (2: Encoding and Retrieval) as independent within-subject variables were run for each EEG frequency band (power density values for the delta, theta, alpha, and beta EEG frequency bands). Type-I errors based on the results of Mauchly’s test, resulting from the non-homogeneity of the variances, were reduced by applying the Greenhouse–Geisser correction to the degrees of freedom when necessary. We further investigated statistically significant main effects using pairwise comparisons. When computing pairwise comparisons, the Bonferroni correction to probability values was used to account for the bias caused by multiple comparisons. To determine the magnitude of significant impacts, partial eta squared (*η^2^*) was estimated. According to Cohen’s norms, effect sizes were classified as small when |10| ≤ *η*^2^ < |0.25|, medium when |0.25| ≤ *η*^2^ < |0.40|, and large when *η*^2^ ≥ |0.40|. The normality of the data distribution was preliminarily assessed by checking kurtosis and asymmetry indices.

Secondly, to explore the relationship between the behavioral indices (EffDec_dec_–i, TolDecCom_dec_–i, and MetaDiff_dec_–i) and the EEG frequency band power (delta, theta, alpha, and beta) derived from the four electrodes (AF7, AF8, TP9, and TP10) during the task phases (Encoding, Retrieval, and Metacognitive), a set of correlational analyses (bivariate Pearson correlational values) were computed. The statistical significance threshold was set at 0.05.

## 3. Results

### 3.1. ANOVA Results

The significant main and interaction effects resulting from the ANOVA test and observed for alpha and beta bands will be reported below. No significant results were observed for delta and theta bands.

#### 3.1.1. Alpha Band

First, a main effect for *Electrode* was found [*F*(1, 12) = 14.985, *p* ≤ 0.001, *η^2^* = 0.577], with lower mean alpha values in TP10 compared to AF7 (*p* ≤ 0.001) and AF8 (*p* = 0.003).

**Figure 2:**
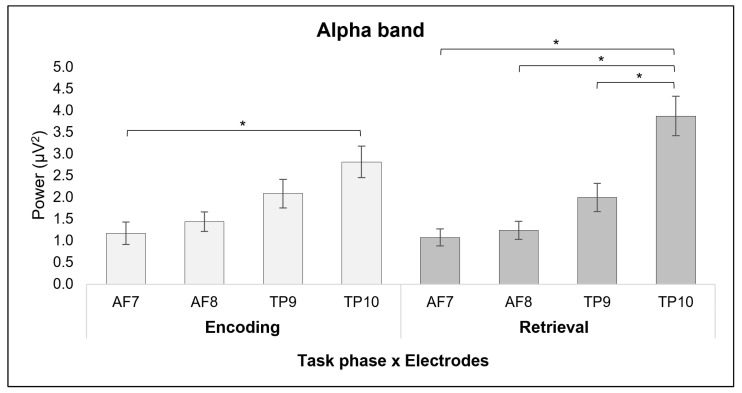
Significant results for the alpha band. The bar graph shows significant differences for the alpha band in different electrode positions during the encoding and the retrieval phases. For all graphs, stars (*) mark statistically significant differences and bars represent ±1 standard error.

Secondly, a significant interaction effect was observed for *Task* × *Electrode* [*F*(1, 12) = 4.405, *p* = 0.010, η*^2^* = 0.286]. Pairwise comparisons showed a decrease in the activation of the TP10 electrode site compared to the AF7 electrode site during the encoding phase (*p* = 0.015). Moreover, during the retrieval phase there was a decrease in the activation of the TP10 electrode site compared to AF7 (*p* ≤ 0.001), to AF8 (*p* ≤ 0.008), and compared to the TP9 (*p* = 0.048) electrode site (Figure 2). No other significant effects were found for the alpha band.

#### 3.1.2. Beta Band

Regarding the beta band, the ANOVA showed a main effect for *Electrode* [*F*(1, 12) = 6.153, *p* ≤ 0.003, η*^2^* = 0.435], with lower mean values in TP10 compared to AF7.

Also, a main effect was found for *Task Phase* [*F*(1, 12) = 12.944, *p* ≤ 0.007, η*^2^* = 0.618], with lower mean beta values in the encoding compared to retrieval phase.

**Figure 3:**
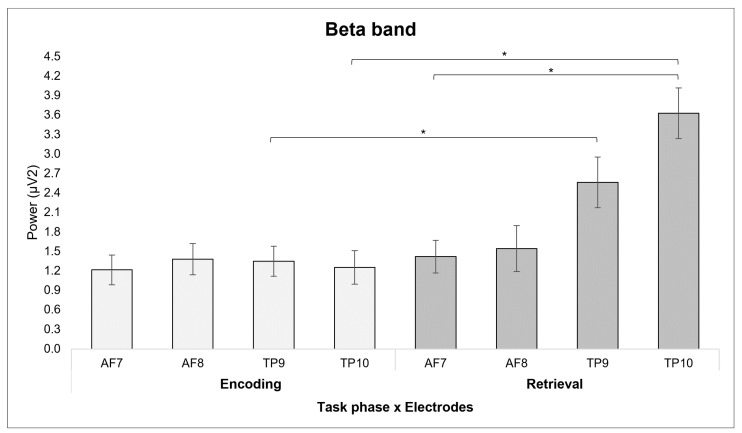
Significant results for the beta band. The bar graph shows significant differences for the beta band in different electrode positions during the encoding and the retrieval phases. For all graphs, stars (*) mark statistically significant differences and bars represent ±1 standard error.

Finally, a significant interaction effect for *Task Phase* × *Electrode* [*F*(1, 12) = 7.223, *p* = 0.001, η*^2^* = 0.474] was found. Pairwise comparisons showed a decrease in activation of the TP9 electrode site (*p* = 0.047) and the TP10 electrode site (*p* ≤ 0.001) during the encoding phase as compared to the retrieval phase. Moreover, during the retrieval phase, there was an increase in the activation of the TP10 electrode site compared to the AF7 electrode site (*p* = 0.001) (Figure 3). No other significant effects were found for the beta band.

### 3.2. Correlational Analysis

**Figure 4:**
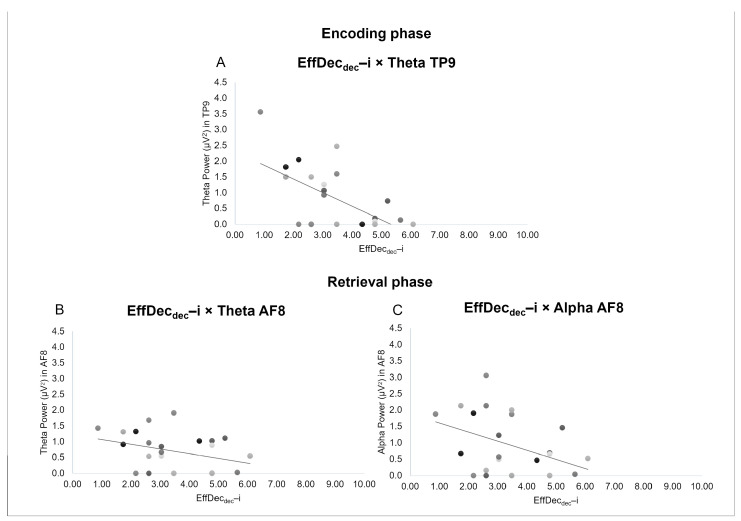
Pearson correlations between the index of Efficiency in complex Decision-making and EEG data. (**A**) The scatter plots display a negative correlation between EffDec_dec_–i obtained during the retrieval phase of the task and the theta band in the TP9 location during the encoding phase and (**B**) AF8 location during the retrieval phase. (**C**) The scatter plots display a negative correlation between EffDec_dec_–i obtained during the retrieval phase of the task and the activity of alpha band in the AF8 electrode during the retrieval phase.

Firstly, the index of *Efficiency in complex Decision-making* (EffDec_dec_–i) obtained during the retrieval phase of the task negatively correlated with the theta band in the TP9 location during the encoding phase (r = −0.809, *p* = 0.000) (Figure 4A), as well as in the AF8 location during the retrieval phase (r = −0.485, *p* = 0.041) (Figure 4B), but also with the activity of the alpha band in the AF8 electrode during the retrieval phase (r = −0.513, *p* = 0.025) (Figure 4C).

**Figure 5:**
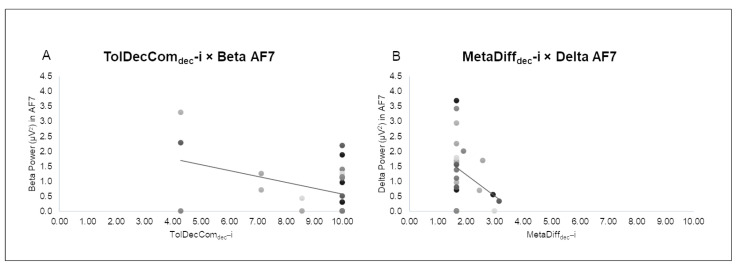
Pearson correlations between the index of Tolerance of Decisional Complexity and EEG data, and the index of Metacognition of Difficulties and EEG data. (**A**) The scatter plots display a negative correlation between TolDecCom_dec_–i obtained during the retrieval phase of the task and the beta band in the AF7 location during the encoding phase. (**B**) The scatter plots display a negative correlation between MetaDiff_dec_–i obtained during the metacognition phase of the task and the delta band in the AF7 location during the retrieval phase.

Secondly, the index of *Tolerance of Decisional Complexity* (TolDecCom_dec_–i) obtained during the retrieval phase of the task negatively correlated with the beta band in the AF7 location during the encoding phase (r = −0.592, *p* = 0.020) (Figure 5A).

Finally, the index of *Metacognition of Difficulties* (MetaDiff_dec_–i) obtained during the metacognition phase of the task negatively correlated with the delta band in the AF7 location during the retrieval phase (r = −0.475, *p* = 0.040) (Figure 5B).

## 4. Discussion

In the present work, we implemented and tested a novel ecological managerial decision-making task that, through the use of professional decision-making scenarios, allows measuring WM workload in relation to contextual variables as well as the gathering of behavioral and EEG data of this process. The task and the procedure were implemented to evaluate the individuals’ capacity to tolerate and respond to circumstances involving increasing decisional complexity (i.e., through the encoding and retrieval phase), but also if they can actively self-represent the difficulties they faced when making decisions (i.e., through the metacognitive phase), both in terms of behavioral and EEG correlates. In fact, to complement behavioral data, this work explored the modulations of EEG cortical oscillations (alpha, beta, delta, and theta frequency bands) related to the cognitive effort during the encoding and retrieval phase of this novel task, but also the relation between behavioral indices—derived from the task performance and computed by calculating individuals’ responses and RTs—and the EEG pattern of brain activity.

Data analysis showed three main sets of results.

### 4.1. The Role of Alpha and Beta Bands in Encoding and Retrieval

First, we observed a decrease in both beta and alpha waves over temporoparietal areas in the encoding phase, when information is loaded into WM. Usually, beta activity is associated with attention and vigilance, often reflecting increased excitatory activity, particularly during states of arousal or focused attention and cognitive processing [29,30,31]. Moreover, beta band activity might be associated with the content representation of WM and integrative functions such as decision-making [49]. Park and colleagues [32] suggest that beta activity is also associated with maintenance of information in WM [31] indicating that beta oscillation might be reflecting a status quo. Also, a decreased beta power was previously associated with high cognitive WM load [48,49]. Alpha, on the other hand, usually indicates the inhibition of processes that are considered conflicting or useless for the task proposed to the individual [24,52], so a decreased alpha activity in a specific area might indicate high cognitive activity [52] and related area activation.

Getting down to specifics, the first result obtained is in line with a recent finding [57,64] showing a bilateral decrease in alpha/beta activity during encoding in the parietal, temporal, and occipital areas, indicating sustained attention and a maintenance process. This pattern, showing lower beta activity within a brain region, is widely thought to reflect the active engagement of that region in current processing [55,65]. Indeed, it has been shown by research combining EEG and fMRI that alpha and beta activity are inversely related to the fMRI blood oxygen level-dependent (BOLD) signal during cognitive processing, mainly reflecting the region activation for the task as well as a load-related effect [41,43,55,66,67,68]. These investigations discovered that lower EEG alpha/beta power in roughly the same location was related to increased BOLD activation.

Proskovec et al. [57] found that the occipital and bilateral cerebellar cortices were engaged in the load-related effects on alpha/beta activity, with load-related differences becoming stronger over time in the right lateral occipital and cerebellar areas, as well as in the left cerebellar cortex. Also, temporoparietal regions have been strongly linked to verbal WM and fundamental language functions [53,54]; specifically during VWM performance, it is believed that the left supramarginal gyrus and posterior temporal regions, overlapping with Wernicke’s area, serve as a temporary storage location for phonological information [53,69,70]. These areas’ activation during VWM might be related to the so-called ‘phonological loop’ [71]—a component of WM—which is essential for speech perception and language comprehension, and it is involved in the short-term storage of speech-based data [72].

Taken together, the results of a decrease in alpha band activity observed in the right temporoparietal area compared to the left frontal area, as well as the decrease in beta band activity in the bilateral temporoparietal areas found in comparison to the retrieval phase, might be related to the activation of regions related to VWM performance—due to the nature of the task—serving as a temporary storage location for phonological information and implicating a load-related effect due to the amount of information presented in the task. However, this pattern of decreased alpha/beta band might also be associated with stress on WM, due to high cognitive load, as suggested by some studies [45,46,48,49] that showed evidence of decreased alpha activity as WM capacity is reached and decreased beta power associated with high cognitive WM load.

Secondly, on the other hand, during the retrieval phase, findings showed a decrease in alpha activity and an increase in beta activity over temporoparietal areas. According to some studies, low alpha activity during the retrieval phase might indicate the retrieval of semantic integrated information [27,55] and active information processing [28,56], while an increase in beta oscillation over posterior areas indicates focused attention and higher cognitive processing [33].

Indeed, several studies showed that as WM task complexity increases, alpha activity decreases in posterior areas [42,58,73] and increases in the frontocentral region [73,74], which might implicate memory gating information [75]. In addition, increasing WM task complexity and high cognitive load seem to also be related to an increase in beta power [46,58] underlying both phase change in the task and filtration of irrelevant information [75,76]. This pattern is prevalent over right parietal areas [46] and might be coherent with a meta-analysis [77] showing how the retrieval phase is strongly associated with the frontoparietal control network, including the left mid-lateral prefrontal cortex (PFC), bilateral anterior insula, bilateral pmPFC, and left inferior intraparietal sulcus regions.

Thus, the strong involvement of the frontoparietal control network in the retrieval phase observed in this study is consistent with the idea that the network underlies EF mechanisms because a retrieval phase almost certainly demands control functions including memory scanning, decision-making, and response selection [78,79,80].

### 4.2. Correlations between EEG Bands and Behavioral Indices

Thirdly, with reference to the association between behavioral indices and EEG cortical activity during this novel managerial decision-making task, we observed a negative correlation between the index of *Efficiency in complex Decision-making* (EffDec_dec_–i) and the theta band over the left temporoparietal area during the encoding phase and over the right frontal area during the retrieval phase. It has been shown that theta increases in frontal areas during WM workload [60]; in particular, it increases as task requirement increases. Also, frontal theta has been associated with a decrease in the default-mode network activity that is linked to increasing cognitive demands [67], and its increase marks the need for cognitive top-down control [81].

Therefore, it might be plausible that the decrease in theta in frontal and left temporoparietal areas may signal a lower need for cognitive control during both the encoding and retrieval phase, and that this is associated with and reflected by an increase in task behavioral efficiency (higher score of EffDec_dec_–i). On the other hand, the increment of theta (an EEG marker that in former studies was demonstrated to increase as task requirement increases) is also associated with lower behavioral efficiency (lower score of EffDec_dec_–i) in the current managerial decision-making task.

Also, a negative correlation has been found between the same index (EffDec_dec_–i) and alpha band activity over the right frontal area during the retrieval phase. This result might indicate, in accordance with the same pattern showed by theta, heightened cognitive activity in frontal areas [52]. Also, a decrease in EEG alpha activity is associated with increased task requirements [42]. The lower presence of the alpha band during the retrieval phase could be due to the increased level of difficulty of the task that required the activation of the right frontal area, which might be related to a greater engagement level and enhanced decision-making abilities [82], and this correlates with greater efficiency in complex decision-making (i.e., to a better ability to correctly allocate employees within the expected time window).

Regarding the index of *Tolerance of Decisional Complexity* (TolCom_dec_–i), a negative correlation was observed between this index and the beta band over frontal left areas during the encoding phase of the task. The increase in beta activity has been previously associated with a state of arousal as well as focused attention and cognitive processing [29,30,31]. Thus, this correlation might be explained by an increased stress level experienced in the encoding phase, and due to the WM task complexity, that has been related to increased beta power [83,84], and in turn, can be associated with a lower tolerance of decisional complexity.

Finally, a negative correlation was found between the index of *Metacognition of Difficulties* (MetaDiff_dec_–i) and delta band over left frontal area during the retrieval phase. Delta has been associated with emotional states [38,39,40] and with motivational processes linked to reward mechanisms, higher levels of emotional engagement, and cognitive processes related to attention [24,37]. Delta increase has been associated with inhibition of the sensory afferences interfering with internal concentration indicating the person’s effort to concentrate and recall their task performance and thus evaluate it objectively [85], and with participants’ internal concentration during WM tasks [86]. For this reason, an increase in delta activity during the retrieval phase might indicate a lower tendency to report the level of difficulty encountered during the task. In this study, no significant correlation was found between the index of *Efficiency in complex Decision-making* (EffDec_dec_–i) and this metacognitive index (MetaDiff_dec_–i), so future studies are necessary to define whether this lower need for metacognitive monitoring is associated with or perhaps due to a greater task performance.

It is important to underline that the results of these correlations should be interpreted with caution, as they might reflect potential patterns of correlation rather than definitive relation; further studies are needed to validate these findings and establish a clearer understanding of their significance.

## 5. Conclusions

To conclude, this study allowed us to explore the behavioral and EEG correlates of healthy individuals performing a novel WM task that strives to measure WM workload in relation to contextual variables, using real-life ecological situations related to managerial decisions. Furthermore, in accordance with recent fMRI studies on working memory [87], which highlighted distinct neural activation during encoding and retrieval phases, our EEG findings similarly demonstrate differential neural engagement during these phases. Specifically, the encoding phase in our study is characterized by decreased alpha and beta activity in temporoparietal regions, indicating increased cognitive load and activation of verbal working memory circuits. During the retrieval phase, the observed increase in beta activity over posterior areas suggests heightened focused attention, aligning with the fMRI findings that implicate memory recall and cognitive control regions during retrieval. This convergence of evidence across methodologies reinforces the distinct neural processes underlying encoding and retrieval, although the spatial resolution differences between EEG and fMRI may lead to some variation in the specific regions identified.

Also, the interrelation between the behavioral performance, the individuals’ ability to consciously self-represent the cognitive and mental processes activated during the task, and EEG correlates have been discussed above.

As a strength point, the realism of the profiles and the ecological features of the situations described in each scenario (e.g., Digitalization, Privacy and data, Market research, Advertising campaigns, Online training, and Team dynamics) can be mentioned; indeed, managers must deal with daily complex situations that call for the integration of contextual factors and the understanding of their employees’ abilities and skills which is necessary to assign them a specific assignment. The stress on the ecological feature of the task was intended firstly because a mere WM task using numbers, letters, or words might not be appropriate for the organizational context and secondly, because we wanted the task to elicit behavioral and EEG responses akin to those that occur in real life.

Despite its innovativeness, it must be noted that this work is not exempt from limitations. Notably, the sample size of this work was relatively small, so behavioral data (such as RTs) might differ in replications, resulting in statistical differences. For these reasons, in the future, further testing and replication with larger cohorts would be desirable in order to strengthen the data interpretation. Also, in the future, it would be interesting to test the validity of this novel task on a sample of selected professionals (such as Human Resources professionals) and managers in a real context.

Furthermore, the advantage of using a wearable EEG device like Muse^TM^ Headband version 2 (InteraXon Inc., Toronto, ON, Canada) also brings some limitations; indeed, given the restricted number of EEG electrodes (AF7, AF8, TP9, and TP10), results and data discussion are reduced to only these scalp areas [88,89]. Thus, future research might look at using multichannel EEG devices to gather more data on brain activity and, in addition, autonomic indices (such as heart rate, heart rate variability, and electrodermal activity) could also be collected to complement neurophysiological data—even if the use of other devices might diminish the procedure ecological validity. Finally, in future research, it would be of interest to investigate if the level of expertise (young versus senior professional), the professional specialty, or individual differences (such as personality traits or cognitive styles) can be associated with a distinct behavioral performance and EEG pattern in the current task.

## Figures and Tables

**Table 1:** Description of three exemplary profiles.

Profile	Description
**Viviana**	Viviana has excellent teamwork skills. She has the ability to work excellently in a team regardless of hierarchical position. She is characterized by enthusiasm and often proposes good initiatives. She is able to create an optimal work environment for production purposes.
**Mauro**	Mauro has excellent digital and technological skills. His strong point is undoubtedly the decision-making process: he is able to excellently evaluate the pros and cons of each alternative, predict the outcomes and choose the one best suited to his objectives.
**Caterina**	Angela has excellent interpersonal skills, knows how to interact, listen, and mitigate conflicts. Characterized by an excellent degree of emotional intelligence, she is able to recognize and manage her own and other people’s emotions to establish better relationships in the workplace. These qualities also make her very qualified in evaluating the skills of others. She is extremely creative and innovative in creating digital, social and educational content.

**Table 2:** Example of the first scenario of the task.

Scenario	Description	Work Assignment
**1—Digitalization**	Your company is launching a new project that aims at digitalizing the hiring interviews. In fact, it is planned to include a phase of simulations and computerized tests within the interviews. To carry out this project, it will be necessary to complete two steps: to design a digital platform and design the most appropriate and creative tests for the evaluation to be included in the platform. Which of your employees would you assign to the following work assignments?	1a. Design of the digital platform1b. Design of creative content

## Data Availability

The data presented in this study are available on request from the corresponding author due to ethical reasons for sensitive personal data protection (requests will be evaluated according to the GDPR—Reg. UE 2016/679 and its ethical guidelines).
